# Innovative Facial Contouring Using a Monopolar Radiofrequency Device with Continuous Water Cooling: An Integrated Clinical and Preclinical Study

**DOI:** 10.3390/ijms27125162

**Published:** 2026-06-06

**Authors:** Hyojin Roh, Young In Lee, Jinyoung Jung, Ngoc Ha Nguyen, Jewan Kaiser Hwang, Jihee Kim

**Affiliations:** 1 Department of Dermatology & Cutaneous Biology Research Institute, Yonsei University College of Medicine, Seoul 03722, Republic of Korea; zinyro@naver.com (H.R.); ylee1124@yuhs.ac (Y.I.L.); nguyenngocha7996@gmail.com (N.H.N.); 2 Mymirae Dermatologic Clinic, Seoul 07326, Republic of Korea; yoracle@naver.com; 3 Scar Laser and Plastic Surgery Center, Yonsei Cancer Hospital, Seoul 03722, Republic of Korea; 4 Department of Dermatology, University of Medicine and Pharmacy at Ho Chi Minh City, Ho Chi Minh City 70000, Vietnam; 5 Mymirae Research Institute for Dermatologic Science, Seoul 07326, Republic of Korea; kaiserwan@hanmail.net; 6 Department of Dermatology, Yongin Severance Hospital, Yonsei University College of Medicine, Yongin-si 16995, Republic of Korea

**Keywords:** monopolar radiofrequency, continuous water cooling, rejuvenation, RF-CWC, extracellular matrix, aging

## Abstract

Monopolar radiofrequency (MRF) is a well-established modality for non-invasive facial rejuvenation; however, its clinical utility is frequently constrained by patient discomfort and inconsistent thermal delivery. This study evaluated the efficacy, safety, and mechanistic profile of a novel MRF system incorporating continuous water cooling (RF-CWC) designed to optimize thermal distribution and enhance patient tolerance. In a prospective, single-arm clinical trial involving 22 female participants, a single RF-CWC treatment utilizing region-specific static and sliding delivery modes yielded statistically significant improvements in jawline lifting, alongside a volumetric increase in the midface and a concomitant volumetric reduction in the lower face (*p* < 0.001) over an 8-week follow-up period, with no adverse events reported. To elucidate the underlying cellular mechanisms, the system was further evaluated using an ultraviolet B (UVB)-induced ex vivo human skin model and an in vivo porcine model. Histological, immunohistochemical, and ELISA analyses revealed that RF-CWC effectively mitigated UVB-induced dermal degradation ex vivo by significantly up-regulating elastin, insulin-like growth factor, and hyaluronic acid, while down-regulating matrix metalloproteinase-1, interleukin-1α, and heat shock protein 72 (*p* < 0.05). Furthermore, the in vivo model demonstrated time-dependent increases in collagen types I and III and elastin without thermal tissue damage, with the sliding mode and higher shot counts correlating with enhanced extracellular matrix (ECM) remodeling. Comparative analyses demonstrated that RF-CWC achieved superior ECM restoration and reduced inflammatory cell infiltration relative to traditional cryogen spray-cooled RF systems. Taken together, these findings suggest that the RF-CWC system may promote robust ECM remodeling and significant facial neocollagenesis while minimizing inflammatory responses, potentially presenting an optimized, highly effective, and patient-friendly advancement in MRF technology.

## 1. Introduction

Skin aging is a multifactorial process influenced by intrinsic factors, such as genetics and metabolism, and extrinsic factors like ultraviolet (UV) radiation, heat, and pollution. These cumulative stressors compromise the skin’s structural and functional integrity, leading to wrinkles, laxity, and dryness—changes that negatively impact appearance and quality of life [1,2]. A key molecular mechanism underlying these changes is the diminished collagen content in the extracellular matrix (ECM) and loss of elasticity, primarily driven by increased activity of matrix metalloproteinases (MMPs) [3]. These molecular alterations are further exacerbated by chronic low-grade inflammation and stress responses, including upregulation of pro-inflammatory cytokines and heat shock proteins, which perpetuate matrix breakdown and impair effective tissue repair [4,5].

With the rise in “prejuvenation”, a proactive approach to delaying visible signs of aging, contemporary aesthetic strategies increasingly emphasize early intervention and long-term skin health preservation [6]. Among the technologies aligned with this preventive philosophy, radiofrequency (RF) has gained prominence due to its multifaceted effects on skin remodeling and rejuvenation. By delivering precisely controlled thermal energy into the dermis and subcutaneous layers, RF triggers a cascade of biological responses that stimulate fibroblast activation and reinforce dermal architecture [7]. Additionally, RF contributes to the reduction in localized adiposity, leading to improved skin firmness, elasticity, and long-term structural resilience—all without compromising the epidermal barrier [8,9]. Since the U.S. FDA approved monopolar RF (MRF) devices for facial wrinkle reduction in 2002, they have continually evolved to optimize both therapeutic efficacy and patient comfort [10].

To prevent RF-induced heat damage, cooling methods are a critical determinant of both safety and treatment performance in RF-based therapies. While RF with cryogen spray cooling (RF-CSC) provides rapid epidermal temperature reduction, its intermittent and localized cooling pattern necessitates static handpiece positioning and may produce heterogeneous thermal profiles within the dermis if the handpiece is detached from the skin [10,11,12,13,14,15,16]. In contrast, RF with continuous water cooling (RF-CWC) offers sustained and uniform epidermal protection during RF energy delivery, enabling stable intradermal heating while permitting dynamic handpiece movement. Specifically, circulating cooling water within the treatment tip dissipates excessive surface heat and maintains epidermal temperature stability throughout treatment, thereby allowing sufficient thermal energy accumulation within the dermal and deeper dermal layers. These features may contribute to improved patient comfort and more uniform heat penetration, thereby enhancing treatment consistency [16].

Previous clinical studies of RF-CWC have demonstrated significant improvements in facial wrinkles, skin roughness, and lifting effects, with enhancements in skin elasticity, density, and volumetric parameters that are comparable to RF-CSC [16,17,18,19]. Notably, RF-CWC has also been reported to achieve better pore reduction compared with RF-CSC, while maintaining favorable tolerability and safety profiles [16]. Despite these encouraging findings, studies directly comparing the static and sliding modes of RF-CWC, conducting histologic evaluations against RF-CSC under standardized conditions, and assessing different energy levels and shot numbers to determine optimal treatment parameters remain limited. Furthermore, the underlying mechanisms and anti-inflammatory effects of RF-CWC have not been sufficiently validated in vivo.

To address these gaps, the present study was designed as an integrated clinical and preclinical investigation of RF-CWC. Clinically, we quantitatively evaluated facial lifting, contouring, and volumetric changes following zone-specific application of static and sliding RF-CWC modes. Mechanistically, ex vivo human skin and an in vivo porcine model were employed to compare RF-CWC with RF-CSC across different energy levels and shot numbers, enabling systematic assessment of ECM remodeling, inflammatory markers, and tissue safety. By combining objective clinical imaging with histological and molecular analyses, this study aims to define mode- and parameter-dependent biological effects of RF-CWC and suggest evidence-based guidance for optimized treatment strategies.

## 2. Results

### 2.1. Clinical Evaluation

**Figure 1:**
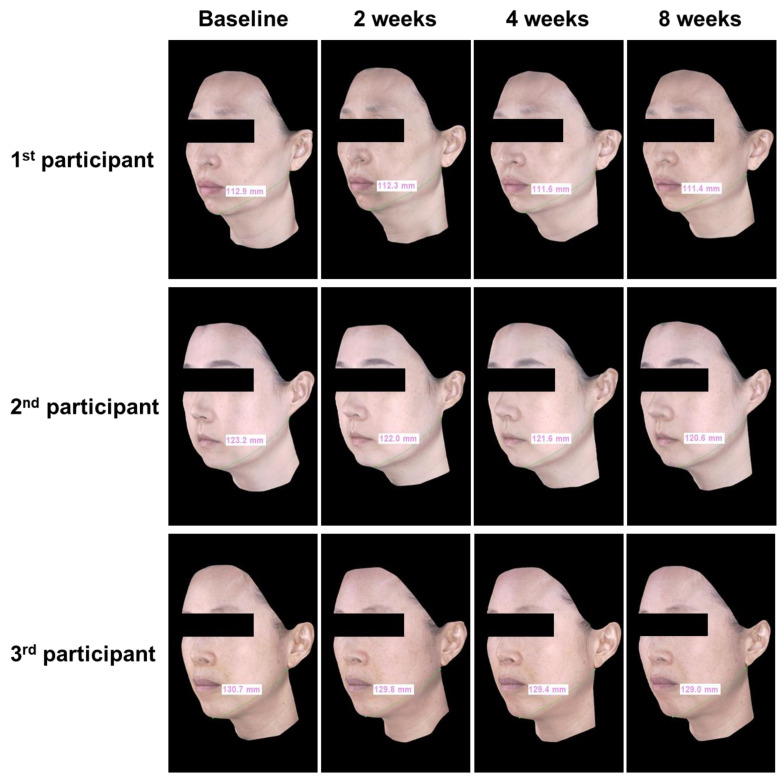
Representative photos of jawline lifting in 3 participants via RF-CWC after 8 weeks. The values demonstrate the green curved distance (mm) from the chin to the earlobe; a decrease indicates a lifting effect of the jawline.

**Figure 2:**
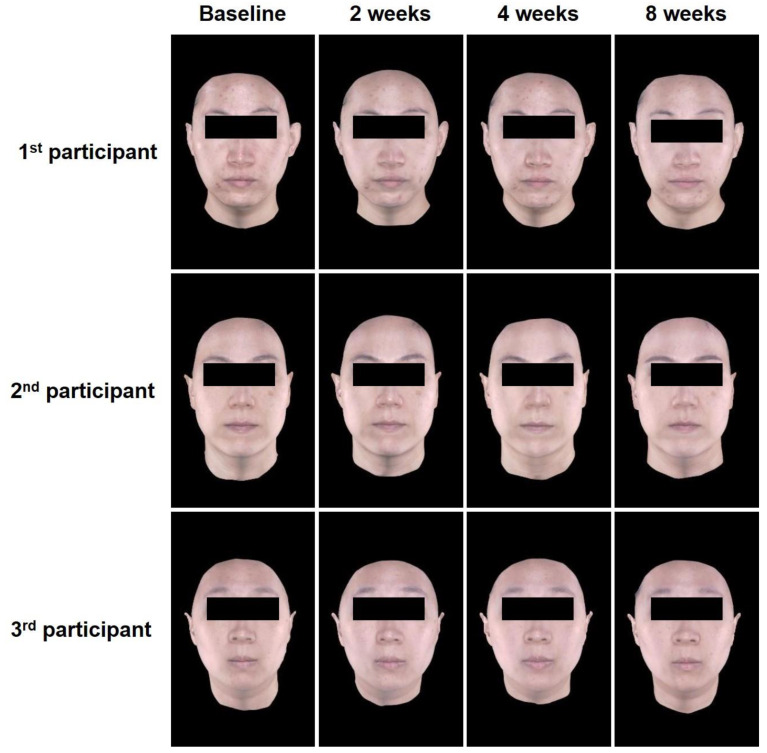
Representative images of changes in central face volume and lateral face contouring in 3 participants via RF-CWC after 8 weeks. After treatment, increased central facial volume and slimmer lateral features were observed.

**Figure 3:**
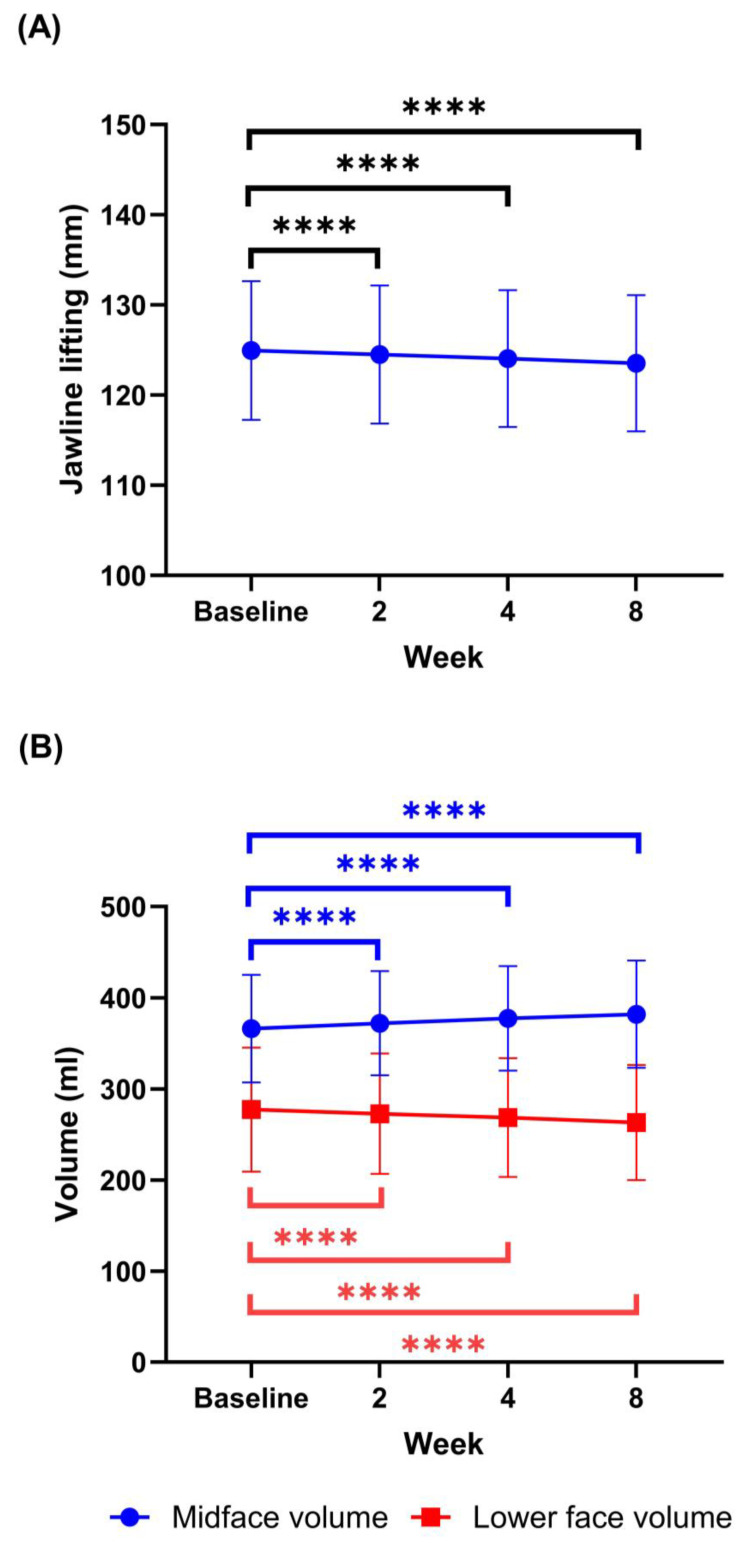
Effects of RF-CWC on the jawline and facial volumes after 8 weeks. (**A**) Jawline lifting (measured as the curved distance from the chin to the earlobe) via RF-CWC after 8 weeks. (**B**) Alterations in midface and lower face volume via RF-CWC after 8 weeks. Comparison with baseline: **** *p* < 0.001.

A total of 22 female participants were enrolled in the prospective, single-arm, clinical study, with a mean age of 44.364 ± 3.374 years. Visual and quantitative assessments consistently demonstrated the lifting and sculpting effects of RF-CWC. Representative images showed noticeable jawline elevation and a more contoured lower face (Figure 1), along with increased central facial volume and slimmer lateral features (Figure 2). These visual improvements were supported by objective data: the curved distance from the chin to the earlobe significantly decreased at visits (V) 1–3 compared to V0 (V0, 124.945 ± 7.682 mm; V1, 124.500 ± 7.658 mm; V2, 124.041 ± 7.585 mm; V3, 123.532 ± 7.548 mm; *p* < 0.001, Figure 3A), and volumetric analysis revealed a significant increase in midface volume (V0, 366.270 ± 59.170 mL; V1, 372.198 ± 57.186 mL; V2, 377.511 ± 57.441 mL; V3, 382.041 ± 58.916 mL; *p* < 0.001, Figure 3B) and decrease in lower face volume over time (V0, 277.330 ± 68.144 mL; V1, 272.789 ± 66.052 mL; V2, 268.742 ± 65.199 mL; V3, 263.090 ± 63.262 mL; *p* < 0.001, Figure 3B). Furthermore, no adverse event was reported in our cohort throughout the trial period. Together, these findings confirm RF-CWC’s dual efficacy and safety profile in facial lifting and contouring.

### 2.2. Ex Vivo Study

**Figure 4:**
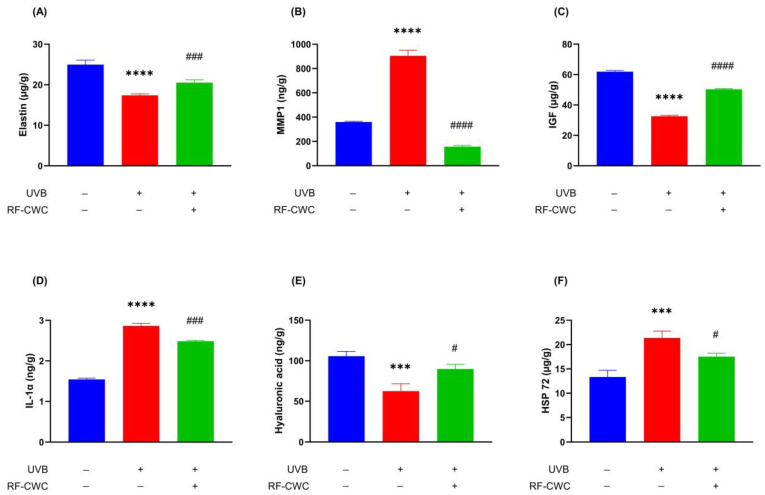
Protein marker expression analysis via ELISA in an UVB-irradiated ex vivo skin model, with or without treatment with RF-CWC. (**A**) Elastin, (**B**) MMP1, (**C**) IGF, (**D**) IL-1α, (**E**) Hyaluronic acid, and (**F**) HSP 72. Comparison with negative control group: *** *p* < 0.005, **** *p* < 0.001. Comparison with UVB-irradiated control group: # *p* < 0.05, ### *p* < 0.005, #### *p* < 0.001. +, treated with UVB or RF-CWC; -, not treated with UVB or RF-CWC. ELISA, Enzyme-Linked Immunosorbent Assay; MMP1, matrix metalloproteinase 1; IGF, insulin-like growth factor; IL, interleukin; HSP 72, heat shock protein 72; UVB, ultraviolet B; RF-CWC, radiofrequency with continuous water cooling.

A UVB-induced ex vivo human skin model was used to assess RF-CWC’s effects on dermal aging. UVB irradiation significantly altered aging-related protein expression, decreasing elastin, insulin-like growth factor (IGF), and hyaluronic acid (HA) while increasing MMP-1, interleukin (IL)-1α, and heat shock protein 72 (HSP 72). RF-CWC treatment effectively reversed these changes, restoring ECM components and reducing inflammatory markers (*p* < 0.05, Figure 4).

### 2.3. In Vivo Study

#### 2.3.1. Histological and Immunohistochemical (IHC) Staining Results

To comprehensively assess the therapeutic effects of RF-CWC, histological and IHC outcomes were compared across treatment parameters, including application mode (static vs. sliding), energy levels (2.5 vs. 4.0), shot numbers, and comparison with RF-CSC.

**Figure 5:**
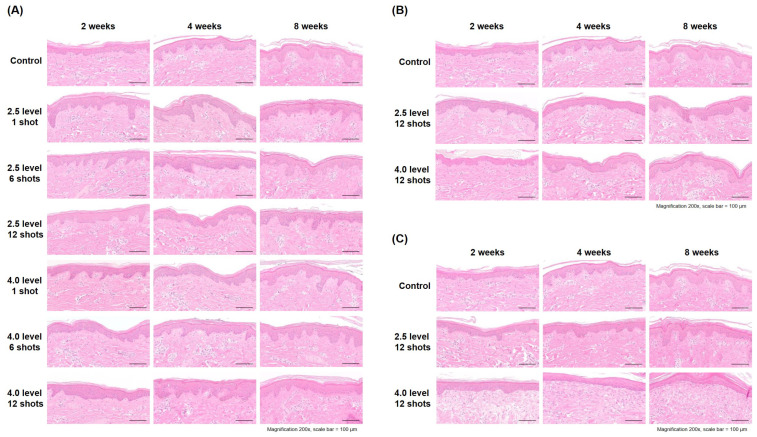
Histopathological changes and tissue remodeling demonstrated via representative hematoxylin and eosin-stained skin sections at 2, 4, and 8 weeks post-treatment of various RF treatment modalities. All treatment parameters safely preserved skin integrity without thermal damage, inducing a prominent increase in collagen deposition that culminated in a denser extracellular matrix and a wavy dermal-epidermal junction. (**A**) RF-CWC static mode stratified by energy level (2.5 and 4.0) and shot count (1, 6, and 12 shots). (**B**) RF-CWC sliding mode (12 shots) stratified by level (2.5 and 4.0). (**C**) RF-CSC static mode (12 shots) stratified by level (2.5 and 4.0). Magnification 200×; scale bar = 100 μm.

**Figure 6:**
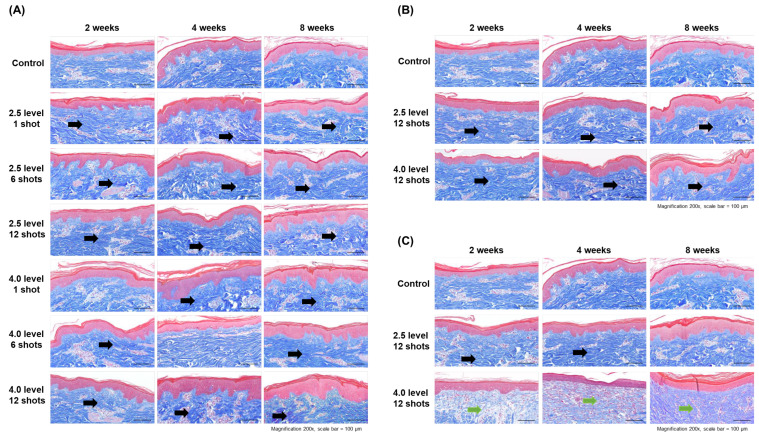
Collagen deposition demonstrated via representative Masson’s trichrome-stained skin sections at 2, 4, and 8 weeks post-treatment of various RF treatment modalities. All treatment parameters induced a prominent increase in blue-stained collagen fiber density. (**A**) RF-CWC static mode stratified by energy level (2.5 and 4.0) and shot count (1, 6, and 12 shots). (**B**) RF-CWC sliding mode (12 shots) stratified by level (2.5 and 4.0). (**C**) RF-CSC static mode (12 shots) stratified by level (2.5 and 4.0). Black arrows indicate localized areas of increased collagen deposition compared to the control skin sections; green arrows indicate areas of dense inflammatory infiltrate. Magnification 200×; scale bar = 100 μm.

**Figure 7:**
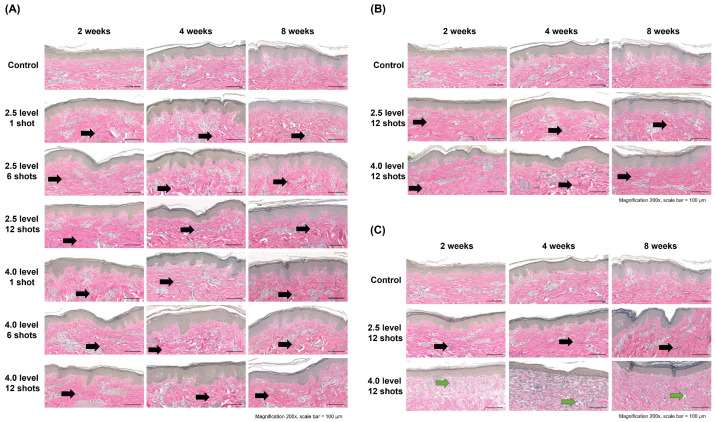
Elastin deposition demonstrated via representative Verhoeff–Van Gieson-stained skin sections at 2, 4, and 8 weeks post-treatment of various RF treatment modalities. All treatment parameters induced a prominent increase in black-stained elastic fiber density. (**A**) RF-CWC static mode stratified by energy level (2.5 and 4.0) and shot count (1, 6, and 12 shots). (**B**) RF-CWC sliding mode (12 shots) stratified by level (2.5 and 4.0). (**C**) RF-CSC static mode (12 shots) stratified by level (2.5 and 4.0). Black arrows indicate localized areas of increased elastin deposition compared to the control skin sections; green arrows indicate areas of dense inflammatory infiltrate. Magnification 200×; scale bar = 100 μm.

Hematoxylin and Eosin (H&E) staining confirmed preserved epidermal and dermal integrity across all groups, with no signs of thermal damage, cellular alteration, or tissue degradation. Over the 8-week period, a significant increase in collagen deposition and dermal remodeling in treated groups was observed compared to the control, characterized by a denser, more organized extracellular matrix. Additionally, treated groups developed a more pronounced wavy pattern at the dermal-epidermal junction alongside a well-maintained epidermis, demonstrating that the treatment successfully stimulated robust dermal regeneration and skin tightening (Figure 5). Representative staining images of Masson’s Trichrome (MT) and Verhoeff–Van Gieson (VVG) staining revealed localized collagen and elastin production over time in all groups (Figure 6 and Figure 7). Notably, RF-CSC at level 4.0 caused a surge in dermal inflammatory infiltrate relative to sparser inflammation cells depicted in RF-CWC static mode at the same level (Figure 5, Figure 6 and Figure 7).

**Figure 8:**
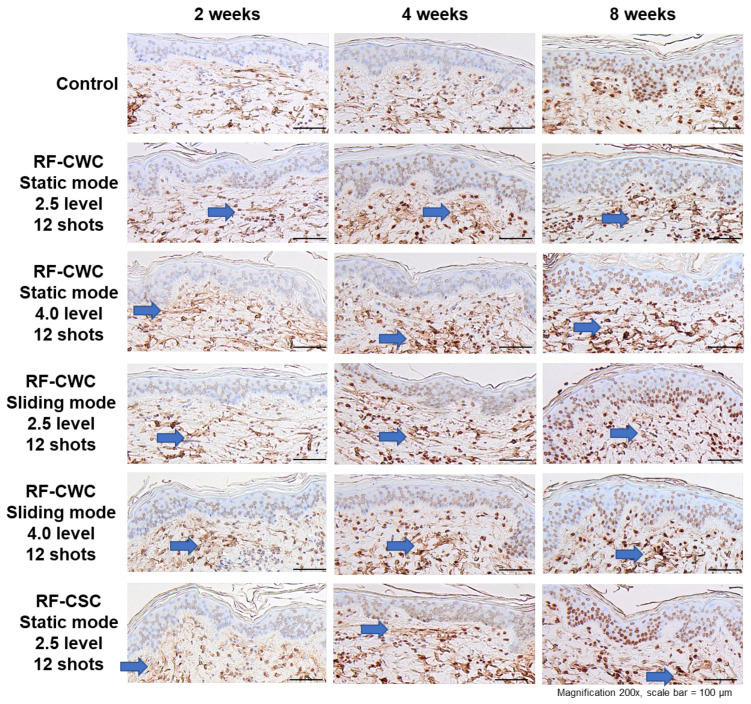
Collagen I expression demonstrated via representative immunohistochemically stained skin sections at 2, 4, and 8 weeks post-treatment of various RF treatment modalities. All treatment parameters induced a prominent increase in collagen I expression. Images display a control versus five specific treatment conditions evaluated at a fixed density of 12 shots: RF-CWC static mode (2.5 and 4.0 levels), RF-CWC sliding mode (2.5 and 4.0 levels), and RF-CSC static mode (2.5 level). Blue arrows indicate localized areas of increased collagen I expression compared to the control skin sections. Magnification 200×; scale bar = 100 μm. RF-CWC, radiofrequency with continuous water cooling; RF-CSC, radiofrequency with cryogen spray cooling.

**Figure 9:**
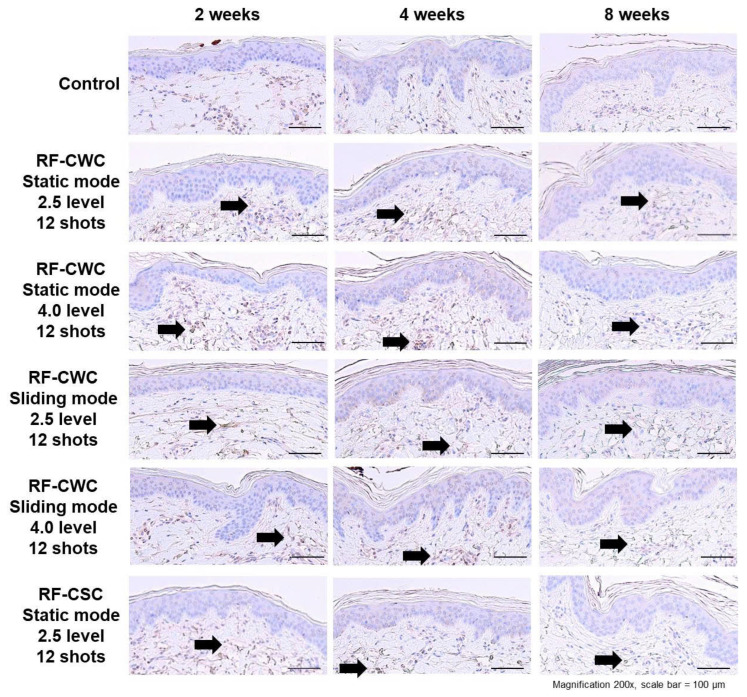
Collagen III expression demonstrated via representative immunohistochemically stained skin sections at 2, 4, and 8 weeks post-treatment of various RF treatment modalities. All treatment parameters induced a prominent increase in collagen III expression. Images display a control versus five specific treatment conditions evaluated at a fixed density of 12 shots: RF-CWC static mode (2.5 and 4.0 levels), RF-CWC sliding mode (2.5 and 4.0 levels), and RF-CSC static mode (2.5 level). Black arrows indicate localized areas of increased collagen III expression compared to the control skin sections. Magnification 200×; scale bar = 100 μm. RF-CWC, radiofrequency with continuous water cooling; RF-CSC, radiofrequency with cryogen spray cooling.

IHC stains for collagen I and III demonstrated increased expression in most RF-CWC-treated groups compared with the control group and levels comparable to those in the RF-CSC group (Figure 8 and Figure 9).

Quantitative analysis of the entire dermal area was performed to support histological observations, comparing collagen and elastin fiber density, as well as collagen I and III expression across treatment groups, as analyzed below:

#### 2.3.2. Comparison Between Static and Sliding Mode of RF-CWC

**Figure 10:**
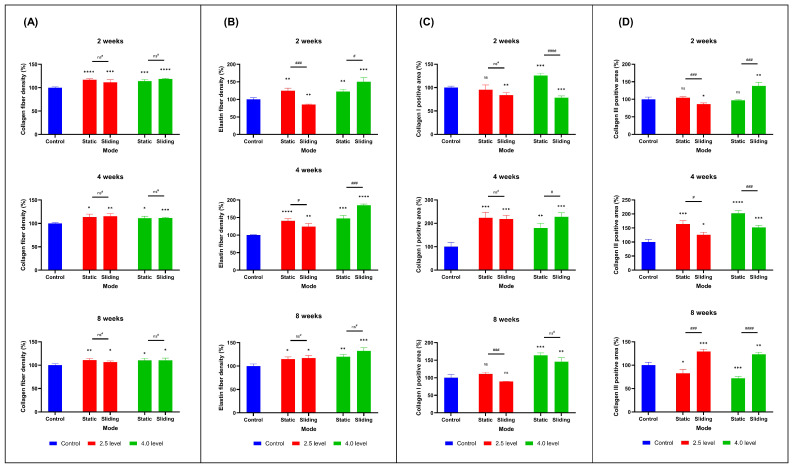
Comparison of different histological measurements between static and sliding mode of RF-CWC at 2, 4, and 8 weeks, using 12 shots. (**A**) Collagen fiber density. (**B**) Elastin fiber density. (**C**) Collagen I positive area. (**D**) Collagen III positive area. Note that the final quantitative results were derived from objective image analyses of the entire dermal area rather than from visual inspection of representative staining images alone. Comparison with control: ns, not significant; * *p* < 0.05; ** *p* < 0.01; *** *p* < 0.005; **** *p* < 0.001. Comparison between static and sliding mode in each level: ns#, not significant; # *p* < 0.05; ### *p* < 0.005; #### *p* < 0.001.

Both modes significantly increased collagen and elastin fiber density using 12 shots at energy levels of 2.5 and 4.0 over 8 weeks (*p* < 0.05, Figure 10A,B), without significant differences between them (*p* > 0.05). However, the sliding mode showed numerically better results in elastin improvement over 8 weeks (*p* > 0.05, Figure 10B). Collagen I expression was significantly elevated at level 4.0 in both modes at week 8 (*p* < 0.05, Figure 10C). Collagen III expression was consistently higher in the sliding mode, increasing over time at both energy levels (*p* < 0.05, Figure 10D).

#### 2.3.3. Comparison Between Different Numbers of Shots via Static Mode RF-CWC

**Figure 11:**
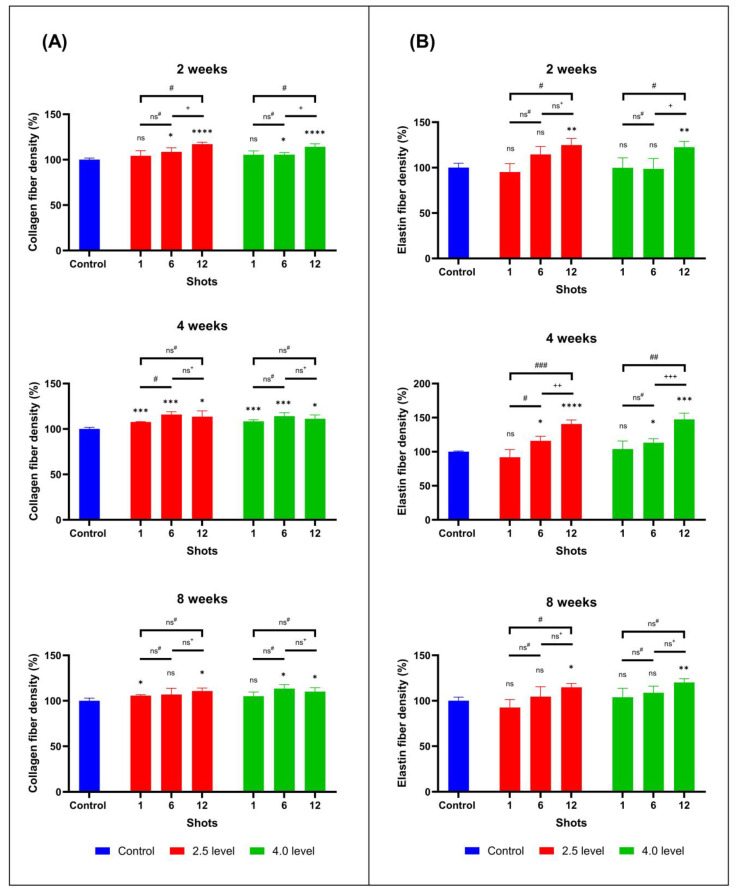
Comparison of collagen and elastin fiber density between different numbers of shots after RF-CWC static mode treatment at 2.5 and 4.0 levels after 2, 4, and 8 weeks. (**A**) Collagen fiber density. (**B**) Elastin fiber density. Note that the final quantitative results were derived from objective image analyses of the entire dermal area rather than from visual inspection of representative staining images alone. Comparison with control: ns, not significant; * *p* < 0.05; ** *p* < 0.01; *** *p* < 0.005; **** *p* < 0.001. Comparison with 1 shot: ns#, not significant; # *p* < 0.05; ## *p* < 0.01; ### *p* < 0.005;. comparison with 6 shots: ns+, not significant; + *p* < 0.05; ++ *p* < 0.01; +++ *p* < 0.005.

Static mode RF-CWC treatment increased collagen fiber density at all time points, with the 12-shot group showing the greatest and earliest enhancement (*p* < 0.05), indicating a dose-dependent and sustained remodeling effect (Figure 11A). Elastin fiber density followed a similar trend, with the 12-shot group consistently outperforming the 1- and 6-shot groups over 8 weeks (Figure 11B).

#### 2.3.4. Comparison Between RF-CWC and RF-CSC

**Figure 12:**
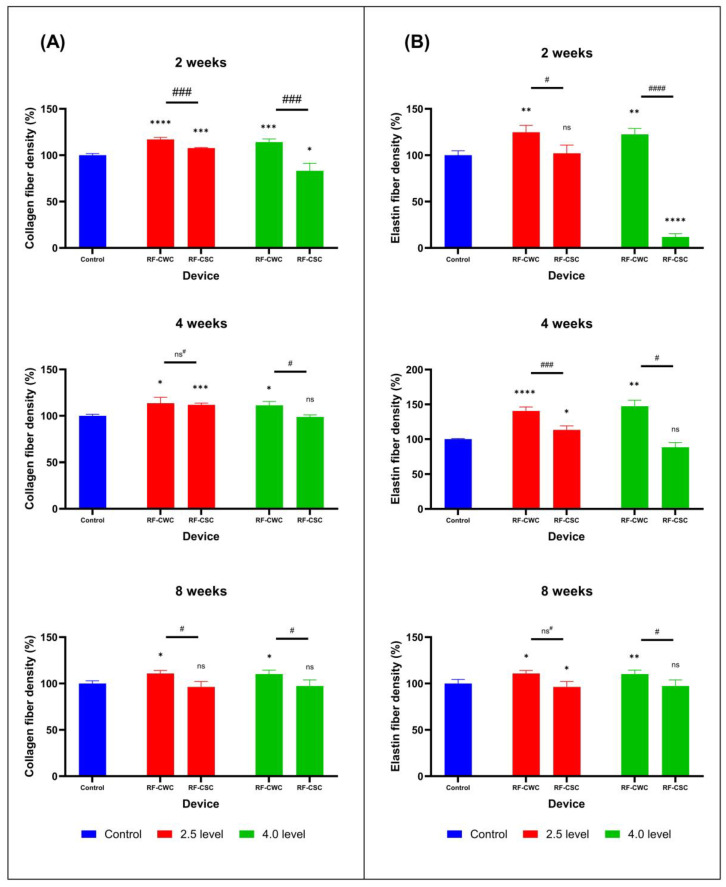
Comparison of different histological measurements between RF-CWC and RF-CSC at 2, 4, and 8 weeks using 12 shots. (**A**) Collagen fiber density. (**B**) Elastin fiber density. Note that the final quantitative results were derived from objective image analyses of the entire dermal area rather than from visual inspection of representative staining images alone. Comparison with control: ns, not significant; * *p* < 0.05; ** *p* < 0.01; *** *p* < 0.005; **** *p* < 0.001. Comparison between RF-CWC and RF-CSC in each level: ns#, not significant; # *p* < 0.05; ### *p* < 0.005; #### *p* < 0.001. RF-CWC, radiofrequency—continuous water cooling; RF-CSC, radiofrequency—cryogen spray cooling.

Both devices significantly increased collagen fiber density compared to the control across all time points and energy levels using 12 shots, with RF-CWC showing consistently higher values than RF-CSC in most conditions (*p* < 0.05, Figure 12A). A similar trend was observed for elastin fiber density, especially at level 4.0 (*p* < 0.05, Figure 12B). Combined with histological observations (Figure 5, Figure 6 and Figure 7), these findings suggest that RF-CWC may deliver better ECM remodelling effects while maintaining a lower degree of treatment-induced inflammation compared to RF-CSC.

#### 2.3.5. Comparison Between Different Energy Levels Across Different Devices, Treatment Modes of RF-CWC, or the Number of Shots of Static Mode RF-CWC

Most measurements showed no significant differences between energy levels 2.5 and 4.0 over 8 weeks when stratified by device (Figure S1), treatment mode of RF-CWC (Figure S2), or number of shots of static mode RF-CWC (Figure S3).

## 3. Discussion

Facial rejuvenation can be achieved through both invasive and non-invasive methods. While surgical lifting, fillers, and threads yield durable results, they carry procedural risks [20,21,22]. Laser-based therapies offer non-invasive alternatives for addressing wrinkles and pigmentation but are limited by shallow tissue penetration and potential adverse effects [23,24]. Recently, MRF has emerged as a safe and effective option across all skin types, offering reliable tightening and lifting [25]. This study evaluated the clinical performance and underlying mechanisms of RF-CWC, a novel MRF device with a CWC system, using clinical, ex vivo, and in vivo models.

As aging occurs, the face undergoes a complex structural transformation driven by a combination of skeletal remodeling, soft tissue redistribution, and cellular degradation [26]. A primary symptom is the loss of midface volume, largely attributed to the atrophy of deep fat pads and the inferior migration of superficial fat pads due to diminished support [27]. In the lower face, aging-induced attenuation of facial retaining ligaments reduces structural tethering, allowing soft tissues to sag inferiorly and anteriorly, which blunts the jawline and contributes to jowl formation [26,28]. The progressive loss of dermal collagen and elastin leads to volume loss and reduced tensile strength and elasticity, thereby making laxity and gravitational deformation more apparent [26,29].

In our clinical study, the sliding mode of RF-CWC delivered a volumizing effect on the midface area prone to age-induced volume loss. Rather than adding artificial bulk, MRF delivers targeted thermal energy to the dermis, stimulating fibroblasts via TGF-β signaling to increase the synthesis of collagen, elastin, glycosaminoglycans, proteoglycans, and growth factors. The accumulation of these ECM components may contribute to enhancing the dermal thickness, volume, and elasticity [30]. Meanwhile, its static mode delivered a lifting effect on the jawline and reduced lower face volume, suggesting that a skin-tightening effect alleviated the sagging commonly associated with this area in aged individuals. While both modes significantly enhanced collagen and elastin deposition, the sliding mode induced greater elastin and collagen III, suggesting suitability for areas requiring volumization, like the midface. In contrast, static mode could be more appropriate for lower facial regions where contour refinement is desired. These volumetric and contouring effects align with previous findings on RF-CWC and other MRF systems [16,19,30,31]. By integrating volumizing and contouring in one session, together with no reported adverse event, RF-CWC enables consistent treatment while maintaining a good safety profile.

Corroborating its clinical lifting effects, RF-CWC demonstrated a multifaceted capacity to reverse photoaging-related damage at the molecular level. The upregulation of collagen I, collagen III, and elastin observed in our preclinical models, which is consistent with previous works [16], suggests structural ECM restoration. Given that skin aging is driven by collagen and elastin degradation via MMPs [32], the concurrent suppression of MMP-1 further highlights the role of RF-CWC in preserving matrix integrity. These findings are consistent with prior evidence demonstrating the ability of RF therapies to promote neocollagenesis and elastin synthesis [33,34,35].

In addition, restoration of HA supported hydration and facilitated the distribution of growth factors and nutrients, while upregulation of IGF promoted cellular growth, metabolism, and tissue repair [36,37,38]. Suppression of IL-1α helped mitigate UVB-induced inflammation and facilitate wound healing, while normalization of HSP 72 expression indicated reduced cellular stress and enhanced protein stability [39,40]. In this context, RF-CWC consistently restored HA and IGF levels while dampening IL-1α and HSP 72 expression in skin tissues following UVB irradiation. Collectively, these findings suggest the translational potential of RF-CWC in addressing the hallmarks of skin senescence by improving both structural integrity and physiological homeostasis. Nevertheless, more detailed mechanistic studies with pathway-level validation are required to substantiate these exploratory findings.

An important contribution of this study is the systematic comparison between shot numbers, devices, and energy levels, which provides practical insight into optimized RF-CWC application. Regarding dose intensity, increasing the number of shots in static mode produced a clear dose-dependent effect, with 12 shots yielding earlier and more sustained ECM remodeling than 1 or 6 shots; thus, higher shot numbers may be preferable for patients with advanced laxity, whereas lower shot numbers may suffice for prejuvenation or maintenance indications. Comparisons between RF-CWC and RF-CSC further demonstrated that RF-CWC achieved better ECM remodeling with reduced inflammatory infiltrates, supporting its use in patients with lower pain tolerance or heightened inflammatory reactivity. Notably, across devices, modes, and shot numbers, energy levels of 2.5 and 4.0 produced largely comparable outcomes, indicating that treatment optimization may rely more on delivery strategy (mode and shot number) than on simply increasing energy. Therefore, individualized treatment protocols tailored to each patient and specific facial region are indispensable.

Compared to topical cosmeceuticals, whose efficacy is highly limited by the stratum corneum and carries risks of local irritation [41], RF-CWC induces equivalent rejuvenation at a deeper structural level while sparing the epidermis. This clinical advantage is further highlighted when contrasting RF-CWC with other energy-based devices. While bipolar and multipolar RF systems may cause minimal pain and downtime compared to MRF, their effects remain highly localized, with a more restricted penetration [42,43]. Resurfacing lasers, while able to induce evident skin renewal, incur more complications at greater treatment depths [44]. Similarly, high-intensity focused ultrasound (HIFU) delivers focused acoustic energy that generates precise thermal coagulation zones in deep tissue. By targeting the deep reticular dermis, superficial musculoaponeurotic system, and platysma while sparing the epidermis, HIFU effectively improves skin laxity through controlled tissue contraction and neocollagenesis. Nevertheless, while HIFU provides a powerful non-invasive lifting alternative, further clinical research and protocol standardization are still required to fully establish its long-term efficacy [45]. Consequently, head-to-head comparative clinical trials are warranted to systematically evaluate the long-term efficacy, safety profiles, and patient tolerance across these distinct energy-based modalities.

A key strength of this study lies in its integrative use of clinical, ex vivo, and in vivo models, enabling a comprehensive, translational evaluation supported by molecular evidence that aligns with histological findings. However, several limitations must be acknowledged. The clinical cohort was characterized by a small sample size, a lack of a treated control group, a relatively short follow-up period, and a homogeneous demographic consisting predominantly of females aged 40 and older, all of which may constrain the generalizability of the findings. Regarding the preclinical models, differences between porcine and human dermal physiology warrant caution when extrapolating results. Furthermore, our laboratory analysis lacks pathway-level investigations and mechanistic validation to fully elucidate the molecular cascades underlying RF-CWC-induced rejuvenation. Additionally, although no visible epidermal thermal injury or adverse skin reactions were observed, skin surface temperature was not objectively monitored during treatment—a parameter that would provide deeper insights into the thermal penetration profile of the device. Consequently, future randomized, double-blind, controlled studies incorporating larger, more diverse patient populations, extended follow-up periods, and pathway-specific mechanistic analyses are warranted. Exploring combination approaches with other modalities may also further optimize therapeutic outcomes.

## 4. Materials and Methods

### 4.1. Clinical Trial

#### 4.1.1. Clinical Study Design and Patient Selection

This prospective single-arm clinical study evaluated the volumizing and contouring effects of RF-CWC (Volnewmer, CLASSYS, Seoul, Republic of Korea) in 22 eligible females who completed the study without dropping out. The protocol was approved by the Clinical Trial Review Board of the Global Medical Research Center (Approval No. GIRB-23614-PM, approval date: 26 June 2023) and registered on clinicaltrials.gov (Identifier: NCT07317089, approval date: 5 January 2026). The study was conducted at the Global Medical Evaluation Academy from July to 13 September 2023, in accordance with the Declaration of Helsinki, and written informed consent was obtained from all participants prior to enrollment.

In compliance with Korea’s Ministry of Food and Drug Safety (MFDS) guidelines (Notification No. 2021-55; 2018 Guideline), at least 20 participants were enrolled to ensure statistical validity for comparative analyses. Eligible participants aged 38–50 years agreed to abstain from any dermatological procedures, including facial lifting, during the study and were able to adhere to the study protocol. Exclusion criteria included pregnancy or breastfeeding, failure to comply with contraception, facial lesions, hypersensitivity, inflammatory or infectious facial conditions, recent use of systemic steroids or phototherapy (within 1 month), recent cosmetic procedures (within 3 months), or any condition deemed unsuitable by the investigator.

#### 4.1.2. Treatment Protocol

Radiofrequency treatment was performed using the RF-CWC device operating at 6.78 MHz with a maximum power output of 115 W. All treatments were performed using a 4 cm^2^ treatment tip (V tip). The system features real-time impedance matching within a range of 75–400 Ω, allowing automatic adjustment of energy output according to tissue resistance, along with additional modulation based on tip contact quality and skin hydration. A continuous water-based contact cooling system is integrated into the handpiece to protect the epidermis while enabling effective dermal heating. The selected energy levels (2.5 and 4.0) were based on the settings most commonly used in actual clinical practice. In addition, the shot numbers were determined to simulate real clinical treatment conditions by calculating the relative exposure area of the experimental tissue compared with the total facial surface area typically treated in humans. Furthermore, different shot conditions were included to evaluate the gradual tissue response and dose-dependent effects according to shot number.

Before treatment, a topical anesthetic cream containing 2.5% lidocaine and 2.5% prilocaine (TaiGuk Pharm Co., Ltd., Hwaseong-si, Republic of Korea) was applied evenly for 40 min. Participants then underwent a single RF-CWC treatment on Day 0 (V0) after eligibility screening and baseline assessments. A region-specific dual-mode approach was employed, with static mode targeting the outer facial regions, including the mandibular and jawline areas. In contrast, sliding mode was applied to the central facial regions. Treatment was delivered at a constant power of 65 W (approximately 65 J per shot, equivalent to power level 2.5). Approximately 300 shots were applied in static mode, while another 300 shots were applied in sliding mode using continuous motion, with around 1000 ms per shot. No additional post-procedure care was required.

Follow-up visits were conducted at weeks 2 (V1), 4 (V2), and 8 (V3). At each visit, participants received facial cleansing followed by a 30 min acclimatization period under controlled conditions (20–24 °C, 45–55% relative humidity). Skin assessments and safety were assessed by recording and analyzing the incidence of adverse events reported across all participants at each follow-up.

#### 4.1.3. Efficacy Evaluations

To objectively quantify topographic changes in facial contours and regional tissue volumes, three-dimensional (3D) facial surface imaging was performed using the Morpheus3D^®^ system (Morpheus Co., Ltd., Yongin-si, Republic of Korea). This system utilizes a high-resolution, light-emitting diode structured-light scanner to capture dense surface topology and reconstruct an accurate computerized polygon mesh model of the patient’s face under standardized lighting and positioning conditions. Anatomical linear distances and volumetric metrics were computed algorithmically via the system’s dedicated clinical simulation software using the following parameters:

- Jawline Lifting Analysis: To evaluate lower face lifting effects, specific anatomical landmarks were plotted on the digital 3D mesh. The software calculated the precise Euclidean curved surface distance (mm) along the mandibular border from the chin to the earlobe. A post-treatment decrease in this linear metric indicated a jawline-lifting effect.
- Regional Volumetric Analysis: Volumetric changes (mL) were determined by digitally aligning and superimposing pre-treatment and post-treatment 3D facial scans via a rigid surface-matching registration algorithm. The software computed the exact spatial volume bounded between the two superimposed surface shells within the midface region (between the eyes and from the philtrum to the nose), where an increase indicated volumization, and within the lower face region (from the philtrum to the chin), where a decrease indicated contouring.

### 4.2. Ex Vivo Study

#### 4.2.1. Ex Vivo Skin Model Preparation and RF-CWC Treatment

Residual human skin samples were collected from a healthy Korean donor who was undergoing breast reconstruction under ethical approval from the Institutional Review Board of Yonsei University College of Medicine, Severance Hospital (IRB No. 4-2023-0635, approval date: 19 August 2023). All experimental procedures were conducted in accordance with the Declaration of Helsinki.

Subcutaneous fat was carefully removed, and the tissues were thoroughly washed multiple times with phosphate-buffered saline (PBS) to eliminate residual impurities. The cleaned tissues were then sectioned into 5 cm × 5 cm (width × length) specimens and randomly assigned to one of three experimental groups: (1) untreated control, (2) UVB exposure only, and (3) RF-CWC treatment followed by UVB exposure.

RF-CWC treatment was administered using the V tip (4 cm^2^), delivering 12 shots per sample at an energy level of 2.5 (16.25 J/cm^2^), with inter-shot intervals of 12 s. Following treatment, the tissues were further sectioned into 1 cm × 1 cm specimens and subjected to UVB irradiation.

UVB irradiation was performed similarly to our previous study using a UV cross-linker (BLX 312; Vilber Lourmat, Collégien, France) [46]. After irradiation, the tissues were maintained on a semi-solid culture medium for 72 h in the same incubating conditions as our previous work [46]. The culture medium was prepared in the same way as our previous studies [47,48].

#### 4.2.2. Enzyme-Linked Immunosorbent Assay (ELISA)

To evaluate dermal matrix remodeling, the ex vivo skin tissues collected 72 h post-treatment were homogenized using a TissueLyser II (Qiagen, Hilden, Germany), centrifuged at 2000× *g* for 10 min, and the supernatant was used for protein analysis. Total protein was quantified using the BCA assay (Sigma-Aldrich, St. Louis, MO, USA) and specific proteins were measured with ELISA kits following manufacturers’ protocols: elastin (Cusabio, Houston, TX, USA), MMP-1 (Abcam, Cambridge, MA, USA), IGF (Abcam), IL-1α (R&D Systems), HA (R&D Systems, Minneapolis, MN, USA), HSP 72 (Biorbyt, Cambridge, UK).

Optical density (OD) was measured using a VARIOSKAN LUX microplate reader (Thermo Fisher Scientific, Waltham, MA, USA), and concentrations were calculated from standard curves.

All the experiments were repeated at least 3 times.

### 4.3. In Vivo Study

#### 4.3.1. Animal Breeding

Male pigs (XP Bio, Anseong, Republic of Korea) were selected due to their anatomical and physiological similarities to humans, including comparable metabolism and organ structure, making them a suitable model for evaluating medical devices. A total of 9 pigs were included in the experiment to balance statistical validity and ethical use of animals, as using too few animals may miss significant effects, whereas excessive numbers raise ethical and resource concerns [49,50]. The protocol received approval from the Institutional Animal Care and Use Committee (IACUC no. BIOSTEP IACUC 23-KE-0274, approval date: 4 September 2023).

This study was conducted in Animal Housing Room No. 1 of HLBIOSTEP Co., Ltd. The animals were housed in a controlled breeding area maintained at 23 ± 3 °C, 55 ± 15% relative humidity, a ventilation rate of 10–20 air changes per hour, a 12 h light–dark cycle, and an illuminance level of 150–300 lux. Environmental parameters—including temperature, humidity, ventilation, and lighting conditions—were regularly monitored throughout the housing period, and no deviations were observed that could have influenced the study outcomes.

Feed for growing pigs (Bodybuilder diet, Daehan Feed Co., Ltd., Incheon, Republic of Korea) was supplied by Dream Bio (Gwangjin-gu, Seoul, Republic of Korea), with approximately 600 g provided in a feeder and made available ad libitum. Drinking water was supplied ad libitum and sterilized using ultraviolet irradiation and microfiltration.

#### 4.3.2. Device Application

**Figure 13:**
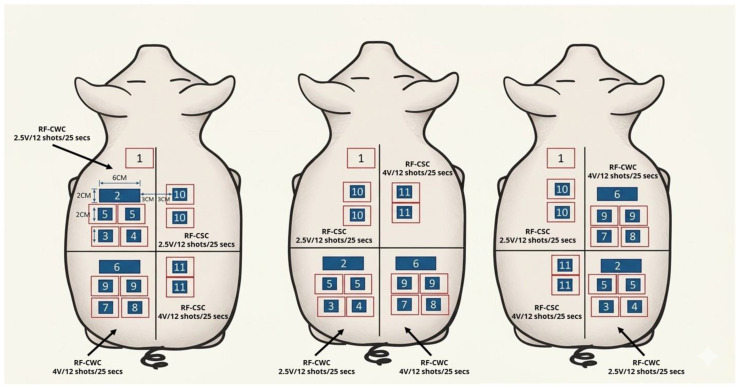
Device Application Sites in Porcine In Vivo Model. Each quadrant on the dorsum of each pig was randomly treated with either RF-CWC or RF-CSC, with smaller areas applied with different treatment parameters (level, number of shots). In total, 11 areas were randomly divided into 11 groups of treatment devices and parameters. The red boxes surrounding the blue rectangles in each quadrant indicate areas treated with the same device, mode, energy level, and area, different from each other by the number of shots, while the blue rectangle not surrounded by the red box is treated with a different mode (see Table 1).

The application conditions for the test device RF-CWC and control device RF-CSC (Thermage FLX, Hayward, CA, USA), including a negative control group, were categorized into 11 treatment conditions based on their mode, energy level, number of shots, and size (Table 1). Pigs were anesthetized and positioned in a ventral recumbent posture. The dorsal region was shaved, disinfected, and treatment sites were marked using a Surgi-pen. To minimize site-specific bias, treatment locations within each group were randomized on the dorsal region of the pigs to prevent the same condition from being applied to the same anatomical area (Figure 13).

Clinical signs, including mortality, were monitored at 1 h intervals for the first 4 h post-treatment and subsequently once daily. At 2, 4, and 8 weeks post-treatment, animals were euthanized under anesthesia via exsanguination through the external jugular vein, and skin tissue samples were collected for analysis.

#### 4.3.3. Histological Analysis

Tissue samples were fixed in 10% neutral-buffered formalin and subsequently embedded in paraffin. Serial sections with a thickness of 5 µm were obtained, mounted onto glass slides, deparaffinized, rehydrated and stained using standard histological techniques: H&E for assessing overall tissue architecture and cell integrity; MT for detecting collagen fibers; and VVG for evaluating elastic fiber remodeling.

- H&E staining: The sections were stained with hematoxylin solution (S3309; Dako, Glostrup, Denmark). After rinsing under running tap water, cytoplasmic staining was performed using eosin solution (318906; Sigma-Aldrich). The stained sections were subsequently dehydrated, cleared, and mounted using a mounting medium. Images were acquired using a digital slide scanner (Aperio A2, Leica Biosystems, Nussloch, Germany), and representative images were captured at 200× magnification using an eSlide viewing software (Aperio ImageScope version 12.4.6; Leica Biosystems).
- MT staining: The sections were mordanted in Bouin’s solution (2010; BBC Biochemical, Mount Vernon, WA, USA) and rinsed under running tap water, followed by nuclear staining with Weigert’s iron hematoxylin (hematoxylin: 4077-4425, Daejung, Siheung, Republic of Korea; ferric chloride: 660, Duksan). Cytoplasm and muscle fibers were subsequently stained with Biebrich scarlet–acid fuchsin solution (Biebrich scarlet: B6008; Sigma-Aldrich; acid fuchsin: 4048-4125; Daejung). The sections were then differentiated and mordanted using a phosphomolybdic–phosphotungstic acid solution (phosphomolybdic acid hydrate: 84235S0410; phosphotungstic acid hydrate: 84220S0410, Junsei, Chuo-ku, Tokyo, Japan). Without intermediate rinsing, collagen fibers were stained with aniline blue (1087-4125, Daejung), and excess dye was removed by treatment with acetic acid. The stained sections were rinsed, dehydrated, cleared, and mounted. Images were acquired using an Aperio A2 slide scanner, and representative images were analyzed at 200× magnification using ImageScope software. Quantitative image analysis was performed using ImageJ software (version 1.54g, NIH, Bethesda, MD, USA) to measure the total area of the papillary dermis and the area occupied by collagen fibers (blue). Collagen fiber density was calculated as the percentage of collagen fiber area relative to the total papillary dermis area.
- VVG staining: The sections were stained with Verhoeff’s solution and differentiated with freshly prepared 2% ferric chloride solution (451649, Sigma-Aldrich), followed by treatment with 5% sodium thiosulfate to remove residual iodine. Counterstaining was performed using Van Gieson solution. The stained sections were dehydrated, cleared, and mounted. Whole-slide images were acquired using the Aperio A2 slide scanner and analyzed at 200× magnification using ImageScope software. Quantitative analysis was performed using Zen Image Analysis software (ZEN 3.4, Carl Zeiss Microscopy GmbH, Jena, Germany) to measure the total papillary dermis area and the area occupied by elastic fibers (black). Elastic fiber density was calculated as the percentage of elastic fiber area relative to the total papillary dermis area.

#### 4.3.4. IHC Staining

Tissue samples collected at 2, 4, and 8 weeks post-treatment were fixed in 10% formalin, embedded in paraffin, and sectioned. After deparaffinization and antigen retrieval, primary antibodies specific to collagen I (MA1-26771, Invitrogen, CA, USA) and collagen III (ab7778, Abcam) were applied, followed by secondary antibodies (anti-mouse: K4001 for collagen I; anti-rabbit: K4003 for collagen III, Dako). Slides were counterstained with hematoxylin (SM806, Dako), mounted, and imaged at 200× magnification using a light microscope (BX43F, Olympus, Tokyo, Japan). Positive staining areas in the dermis were quantified using ImageJ, with greater positive areas indicating higher protein expression. The expression of collagen I and III was evaluated specifically within the dermal region, where collagen fibers are primarily distributed, and quantitative analysis was performed by defining the dermal area as the region of interest. The epidermal region and nuclear staining patterns were not included in the quantitative assessment.

All the experiments were repeated at least 3 times.

### 4.4. Statistical Analysis

Statistical analyses were performed using IBM SPSS Statistics (version 27.0, IBM Corp., Armonk, NY, USA), with significance set at *p* < 0.05. Graphs were created using GraphPad Prism (version 10.2.2; GraphPad Software, Boston, MA 02110, USA). In the in vivo and ex vivo studies, normality was assessed prior to between-group comparisons, followed by either an independent *t*-test for parametric values or a Mann–Whitney test for non-parametric values. For within-group comparisons over time in the clinical study, a repeated-measures ANOVA or the Friedman test was used based on normality, followed by a post hoc analysis using the Wilcoxon signed-rank test with Bonferroni correction.

## 5. Conclusions

RF-CWC, a novel MRF device with a CWC system, demonstrated safe and effective facial rejuvenation through zone-specific application of static and sliding modes. Preclinical data confirmed its ability to remodel the ECM, reduce inflammation, and enhance dermal stability. Compared to conventional systems, RF-CWC might offer better clinical flexibility and safety. Future randomized, double-blind, controlled trials incorporating diverse populations and longer follow-up are needed to confirm its therapeutic potential.

## Figures and Tables

**Table 1:** Device Parameters for different groups of treatment.

Condition	Device	Mode	Energy (Level)	Shots	Size
1	–	–	–	–	–
2	RF-CWC	Sliding	2.5	12	2 × 3 cm
3		Static	2.5	1	2 × 2 cm
4		Static	2.5	6	2 × 2 cm
5		Static	2.5	12	2 × 2 cm
6		Sliding	4.0	12	2 × 3 cm
7		Static	4.0	1	2 × 2 cm
8		Static	4.0	6	2 × 2 cm
9		Static	4.0	12	2 × 2 cm
10	RF-CSC	Static	2.5	12	2 × 2 cm
11		Static	4.0	12	2 × 2 cm

## Data Availability

The original contributions presented in this study are included in the article/Supplementary Material. Further inquiries can be directed to the corresponding author.

## Supplementary Materials

The following supporting information can be downloaded at: https://www.mdpi.com/article/10.3390/ijms27125162/s1.

## Abbreviations

The following abbreviations are used in this manuscript:

ECM	Extracellular matrix
ELISA	Enzyme-linked immunosorbent assay
HA	Hyaluronic acid
H&E	Hematoxylin and eosin
HSP 72	Heat shock protein 72
IGF	Insulin-like growth factor
IHC	Immunohistochemistry
IL	Interleukin
IL-1α	Interleukin-1 alpha
MMP-1	Matrix metalloproteinase-1
MRF	Monopolar radiofrequency
MT	Masson’s trichrome
PBS	Phosphate-buffered saline
RF	Radiofrequency
RF-CSC	Radiofrequency with cryogen spray cooling
RF-CWC	Radiofrequency with continuous water cooling
UV	Ultraviolet
UVB	Ultraviolet B
VVG	Verhoeff–Van Gieson
