# Supplementary material for: Innovative Facial Contouring Using a Monopolar Radiofrequency Device with Continuous Water Cooling: An Integrated Clinical and Preclinical Study
Source: Int J Mol Sci. 2026 Jun 6;27(12):5162. doi: 10.3390/ijms27125162 (PMC13299460; doi:10.3390/ijms27125162)
Supplement: Supplementary file 1 [file ijms-27-05162-s001.zip › ijms-4349962-supplementary.pdf]

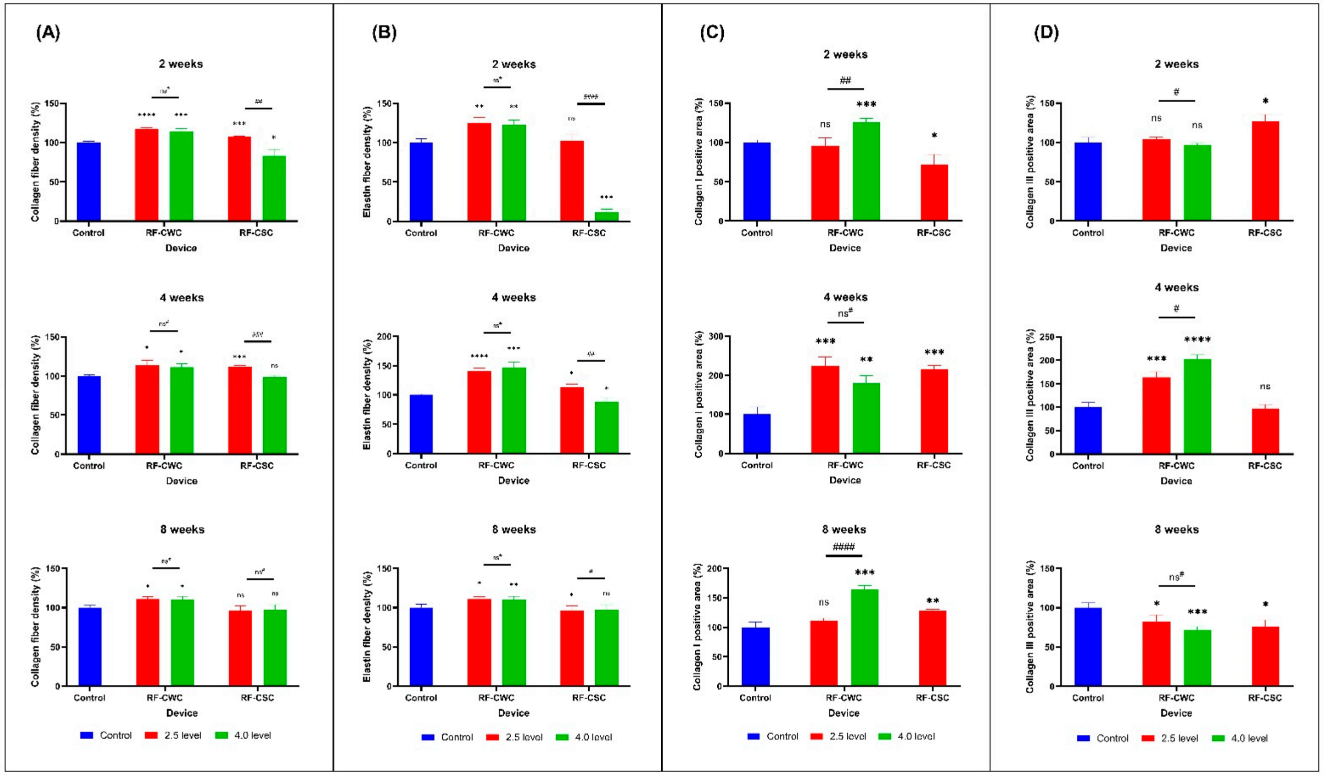

Figure S1. Comparison of different measurements between 2.5 and 4.0 levels at 2, 4, and 8 weeks, stratified by type of device, using 12 shots. (A) Collagen fiber density. (B) Elastin fiber density. (C) Collagen I positive area. (D) Collagen III positive area. *comparison with control; ns, not significant; *$p < 0.05$; **$p < 0.01$; ***$p < 0.005$; ****$p < 0.001$. $^{\#}$comparison between 2.5 and 4.0 levels; ns$^{\#}$, not significant; $^{\#}p < 0.05$; $^{\#\#}p < 0.01$; $^{\#\#\#}p < 0.005$; $^{\#\#\#\#}p < 0.001$.

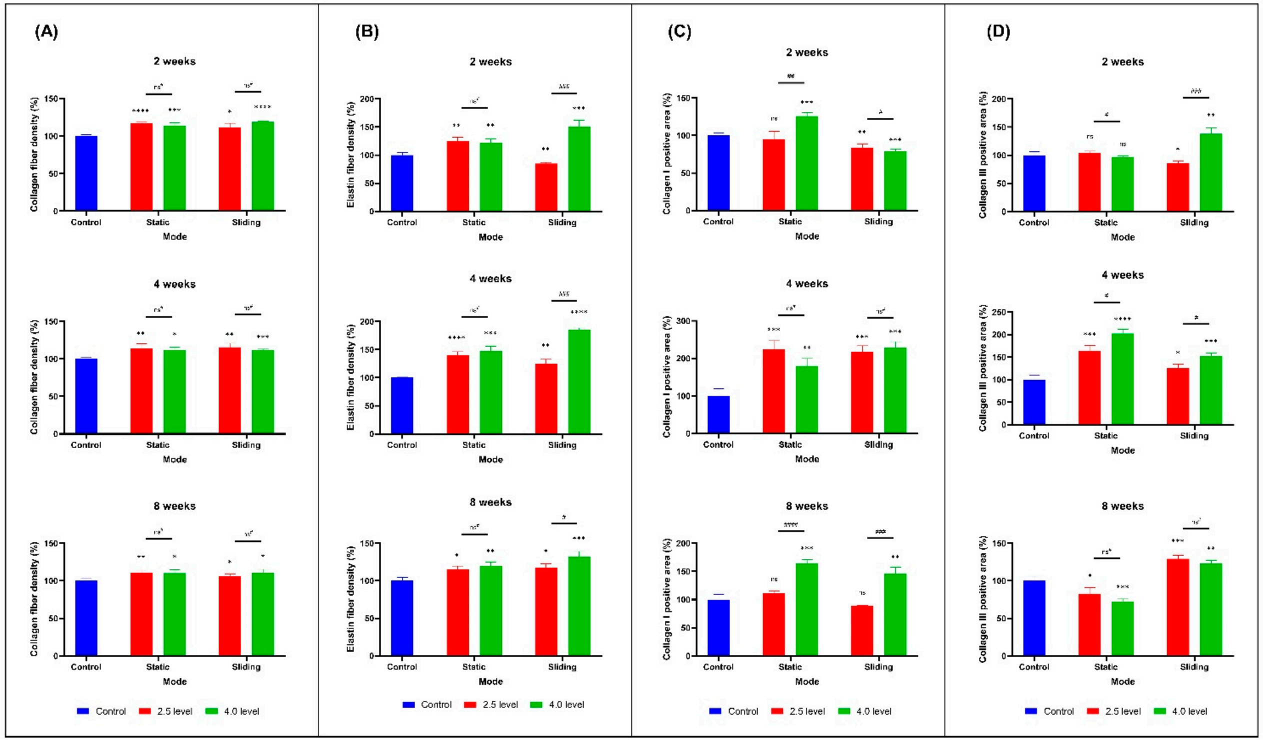

Figure S2. Comparison of different measurements between 2.5 and 4.0 levels at 2, 4, and 8 weeks, stratified by treatment mode of RF-CWC, using 12 shots. (A) Collagen fiber density. (B) Elastin fiber density. (C) Collagen I positive area. (D) Collagen III positive area. *comparison with control; ns, not significant; *$p < 0.05$; **$p < 0.01$; ***$p < 0.005$; ****$p < 0.001$. $^{\#}$comparison between 2.5 and 4.0 levels; ns$^{\#}$, not significant; $^{\#}p < 0.05$; $^{\#\#}p < 0.01$; $^{\#\#\#}p < 0.005$; $^{\#\#\#\#}p < 0.001$.

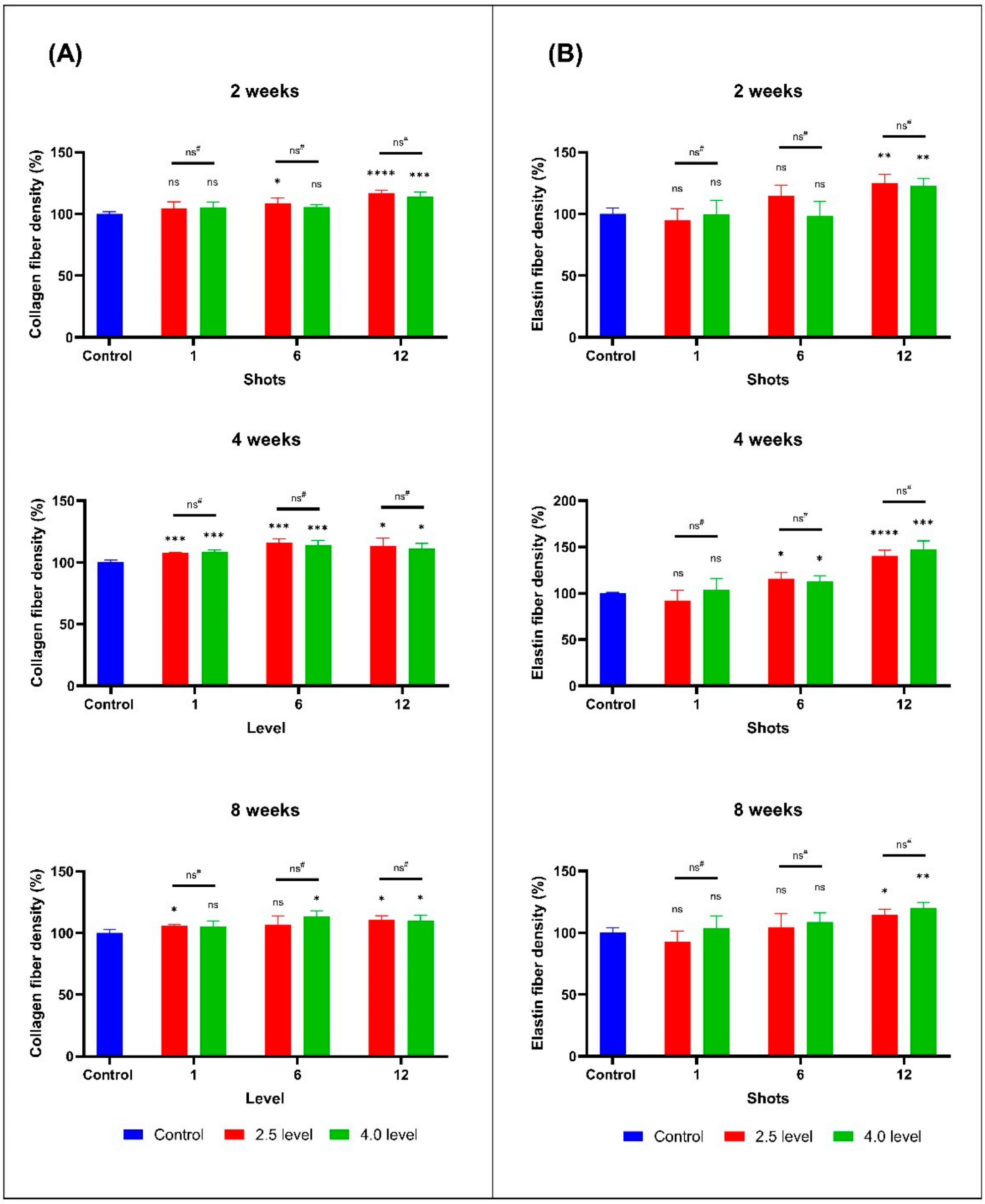

Figure S3. Comparison between 2.5 and 4.0 levels at 2, 4, and 8 weeks, stratified by the number of shots of static mode RF-CWC. (A) Collagen fiber density. (B) Elastin fiber density. (C) Collagen I positive area. (D) Collagen III positive area. (E) HSP 72 positive area. *comparison with control; ns, not significant; *$p < 0.05$; **$p < 0.01$; ***$p < 0.005$; ****$p < 0.001$. $^{\#}$comparison between 2.5 and 4.0 levels; ns$^{\#}$, not significant; $^{\#}p < 0.05$; $^{\#\#}p < 0.01$; $^{\#\#\#}p < 0.005$; $^{\#\#\#\#}p < 0.001$.
